# The Sydney playground project- levelling the playing field: a cluster trial of a primary school-based intervention aiming to promote manageable risk-taking in children with disability

**DOI:** 10.1186/s12889-015-2452-4

**Published:** 2015-11-14

**Authors:** Anita C. Bundy, Shirley Wyver, Kassia S. Beetham, Jo Ragen, Geraldine Naughton, Paul Tranter, Richard Norman, Michelle Villeneuve, Grace Spencer, Anne Honey, Judith Simpson, Louise Baur, Julia Sterman

**Affiliations:** Faculty of Health Sciences, the University of Sydney, Sydney, Australia; Institute of Early Childhood, Macquarie University, Sydney, Australia; School of Exercise Science, Australian Catholic University, Locked Bag 2002, Strathfield, NSW 2135 Australia; School of Physical, Environmental and Mathematical Sciences, the University of New South Wales, Canberra, Australia; Centre for Health Economics Research and Evaluation, University of Technology, Sydney, Australia; School of Public Health, the University of Sydney, Sydney, Australia; The University of Sydney, The Children’s Hospital Westmead, Sydney, Australia

**Keywords:** Occupation therapy, Children, Play, Disability

## Abstract

**Background:**

Providing children and adults with opportunities to engage in manageable risk taking may be a stepping stone toward closing the gap in life conditions currently experienced by young people with disabilities. We aim to demonstrate the effectiveness of a simple, innovative program for 1) changing the way parents and teachers view manageable risk-taking for children with disabilities and 2) increasing the level of responsibility that children take for their own actions, as seen on the school playground.

**Methods/Design:**

We will employ a cluster repeated measures trial with six Sydney-area primary-school-based programs for children with disabilities. The intervention comprises two arms. 1) Risk-reframing- teachers and parents will participate together in small group intervention sessions focusing on the benefits of manageable risk-taking; 2) Introduction of play materials- materials without a defined purpose and facilitative of social cooperation will be introduced to the school playground for children to use at all break times. A control period will be undertaken first for two school terms, followed by two terms of the intervention period. Outcome measures will include playground observations, The Coping Inventory, qualitative field notes, and The Tolerance of Risk in Play Scale.

**Discussion:**

New national programs, such as Australia’s National Disability Insurance Scheme, will place increasing demands on young people with disabilities to assume responsibility for difficult decisions regarding procuring services. Innovative approaches, commencing early in life, are required to prepare young people and their carers for this level of responsibility. This research offers innovative intervention strategies for promoting autonomy in children with disabilities and their carers.

**Trial Registration:**

Australian and New Zealand Clinical Trials Registration Number ACTRN12614000549628 (registered 22/5/2014).

## Background

Having heard the appeals of people with disabilities for greater choice and control in everyday life, Australian governments, both federal and state, have taken action. Government commitment is seen in the National Disability Strategy, the National Disability Insurance Scheme and the New South Wales Disability Support Reform. The ultimate goal of all of these initiatives is to support people with a disability to maximise their potential and participate as equal citizens. Despite these efforts, Australia remains a long way from meeting that goal. Indeed, in early 2013, by considering Australia’s Social Inclusion Indicators Framework [1] together with Household Income and Labour Dynamics in Australia data, Emerson et al. [2] revealed unequivocally that young Australians with a disability are five times more likely than non-disabled peers to experience multiple and entrenched disadvantage in resources and participation. Moreover, despite services and policies ostensibly addressing those needs, the gap is widening.

### The cause

Poor life conditions experienced by many people with disabilities were once thought to be an inevitable consequence of impairment. However, in recent decades, the negative impacts of discrimination and environmental factors on quality of life have become apparent [3–5]. Discrimination is associated with widespread negative beliefs and attitudes [6]. More than half of submissions to the National Disability Strategy Consultation Report, Shut Out [6], identified social attitudes as significant barriers to achieving equality or life satisfaction. Negative attitudes are so entrenched in Australian society that they are often expressed by people with disabilities themselves and by their families. Furthermore, discrimination from potential employers is rampant. Our own work [7], for example, yielded widespread comment that employers do not hire teens or adults with disability because, “they won’t get a decent day’s work for the wage”.

### Beating the odds

Every year, more than 20,000 children in Australia are born with a disability. Most will not “grow out of it” and they will not be cured. Despite the odds, many of those children will be employed and achieve great success. What sets those people apart from the majority? Kurt Fearnley, paralympian and 2013 Australian of the Year, attributed an important measure of his success to positive environmental factors-- his family, teachers, neighbours and friends who “never told me what I couldn’t do. Instead they sat back and found out what was possible [8]”.

### How do children earn respect?

Typically-developing children earn self-respect and the respect of others in an age-related cycle that involves meeting increasingly greater responsibilities [9]. Older children, and children deemed capable, earn the right to take on more complex responsibilities (i.e., where failure has more significant consequences). Disruption to the cycle of “proving oneself” by taking on greater responsibility is an unfortunate and unintended consequence of the increasing desire of some adults to protect children from all risk (i.e., risk aversion, surplus safety). The cycle is even more likely to be disrupted for children with disabilities, given the commonly-held perception that they are less capable and more in need of protection than other children [6, 10]. As Fernley indicated, “Growing up with a disability doesn’t bring with it a sense of shame or self-doubt; it’s only when we learn to interpret the faces of the people around us, or when our environment offers no chance of interacting on an ordinary level, that we learn such things [8]”.

### Sydney Playground Project (SPP): a cluster randomised trial

In 2008 and 2009, our team published findings from a pilot study on children’s playground activities [11, 12]. Subsequently, in 2011 we completed a 3-year National Health and Medical Research Council and Australia Research Council funded trial [13] to test the effectiveness of the two programs proposed here: 1) risk re-framing workshops targeting beliefs of adults and 2) introduction of loose materials with no obvious play value to the school playground. The aim of this study was to increase social skills and physical activity of typically developing children and alter perceptions held by their parents and teachers. The findings from this study suggested that this methodology for addressing the above aims was both feasible and appropriate. Our qualitative findings revealed that adults enjoyed thinking about what children can do rather than what they should not do; that they came to understand the benefits of manageable risk (e.g., taking responsibility) and that they learned new strategies for promoting manageable risk-taking. We observed, and heard about, children engaging in wonderfully creative, social and active play. Quantitative findings revealed increases in physical activity and decreases in sedentary behaviour. We discuss relevant details and findings from the two programs immediately below.

### Our project addresses an important problem

Approximately 9 % of all Australian children between the ages of 5 and 14 years are reported to have a disability [14]. It seems pertinent to suggest that the wellbeing of all children with disabilities is at risk. As they grow up, they are five times more likely than peers without disability to feel unsupported by family and friends, drop out of school, develop mental illness and fail to find employment [3]. Despite Australia’s ratification of the United Nations Convention on the Rights of Persons with Disabilities and the development of policies and services related to disability, the gaps in participation and resources between young people with disability and their typically developing peers continue to widen.

### Innovative ideas underpin the research

We aim to demonstrate the effectiveness of simple, cost-effective interventions for changing the way parents and teachers view manageable risk-taking for children with disabilities. Consequently, we also aim to demonstrate increases in the level of responsibility children take for their own actions.

### Innovative interventions are the heart of the research

Most interventions for parents and teachers of children with disabilities focus on ameliorating deficits by teaching specific programs. We take a strengths-based approach, creating opportunities for parents and teachers to work together to clarify long-term priorities and create new strategies to meet those goals. Based on our previous work [15], we expect that parents and teachers will identify resilience, autonomy and taking responsibility as attributes that children with disabilities need in order to become productive and happy adults. With these attributes as a starting point, we expect parents and teachers to rethink the kinds of experiences they create for, and with, children.

Most professionals who conduct child-based play interventions use play as a medium for teaching skills. As such, adults decide how the play will look. Conversely, we propose to set up the environment in such a way that children determine the play. The play will be the medium for learning whatever children choose but importantly, it will always yield genuine consequences. Playground staff will ensure that nothing truly dangerous happens; they will have many naturally occurring opportunities to help children take responsibility. We anticipate staff will use more problem solving questions than directives (e.g., what will happen if you . . . ? how could you . . . ?), rather than hard and fast rules (e.g., no running!), will set the play boundaries. Other researchers [16] have reported on the use of recycled materials on the playground to promote psychosocial outcomes. However, none have presented the outcomes associated with implementation of recycled materials on the playground in conjunction with a risk reframing program for adults.

We aim to demonstrate the effectiveness of a simple, innovative program for 1) changing the way parents and teachers view manageable risk-taking for children with disabilities and 2) increasing the level of responsibility that children take for their own actions, as seen on the school playground. This project represents a radically different approach for reducing social exclusion of children with disabilities. The research builds on our previous work targeting typically developing children, their parents and teachers. It is hypothesised that 1) the risk re-framing workshops will help parents and teachers distinguish manageable from unhealthy risk and recognize the benefits of manageable risk taking (e.g. becoming more responsible and vigilant) and 2) introducing large loose materials with no obvious play value to the school playground will provide opportunities for adults and children to practice promoting and engaging in manageable risk-taking in the context of social, creative and active play.

## Methods/Design

### Overall study design

We will employ a cluster repeated measures trial with six Sydney-area primary-school-based programs for children with disabilities. The control period will be undertaken first for two school terms, followed by two terms of the intervention period.

All of the ~300 children enrolled in the six programs, their teachers and parents will participate. Table 1 outlines the timeline for the study. All participating schools have agreed to have numbered children (by means of a numbered sticker on the back of their shirts) and support the workshops and inventories. Schools will continue with normal recess and physical education. There are no exclusion criteria, however video, Coping Inventory data and identified observations will only be collected from consenting children. Staff will also provide consent to participate in the study and complete the Coping Inventories and Adult Playfulness Trait Scale (APTS).

**Table 1:** Outcome variables in relation to hypotheses

Outcome Variables	Hypotheses: Significant increases in:
Adults’ promotion of manageable risk taking and children’s increasingly responsible actions on the playground	Ratio of time spent in positive play transactions^a^ to time spent in negative transactions^b^ (primary measure derived from modified SOPLAY described below).
Perceptions of parents and teachers regarding manageable risk taking and children’s abilities to assume responsibility	Positive opinions and stories expressed in interview
Child coping ability	Mean scores on the Coping Inventory^23^
Parents’ and teachers’ comfort with children taking manageable risks	Mean scores on the Tolerance of Risk in Play Scale (TRiPS)^15^ and a revised Willingness to Grant Autonomy Scale^21^

^a^adults may facilitate or join the play

^b^(e.g., aimless wandering or where adults stop or redirect)

### Ethics

Written informed consent will be received from all schools and staff involved in the study. The research is performed in accordance with the ethical standards of the University and the revised (2000) Helsinki Declaration. Ethical approval was obtained from the Human Research Ethics Committee at The University of Sydney (2014/155). This trial has been registered with the Australian and New Zealand Clinical Trial Registry (#ACTRN12614000549628). All participating children will have informed consents signed by their parents prior to their involvement in data collection.

### Sample size

The sample size calculation involved deciding on the number of programs (clusters) rather than the number of children per school (we will include all children in each program who are accessing the playground with the additional materials). The desired number of schools is one that provides sufficient cluster-level data, at the same time, is manageable in terms of implementation and data collection. We have selected six programs, the minimum number recommended for repeated measures cluster trials.

### Intervention phase

The intervention comprises two arms. 1) Risk-reframing- teachers and parents will participate together in small group intervention sessions focusing on the benefits of manageable risk-taking (including vigilance and taking increasingly greater responsibility for one’s own actions), the consequences of preventing children from engaging in manageable risk-taking, and strategies for making everyday risks manageable. Each program will engage in three two-hour sessions during the intervention phase. The first session will coincide with the introduction of the play materials. The second session will follow approximately 3–4 weeks after the first session. The third session will be implemented in the final weeks of the intervention period. Sessions will be scheduled to maximise attendance and multiple sessions in each school may be required. 2) Introduction of play materials. Materials without defined purposes, which are also considered facilitative of social cooperation, will be introduced to the school playground for children to use at all break times. Materials will conform to Australian standards and meet seven additional criteria: 1) no obvious play value; 2) encourage cooperative, gross motor play; 3) have multiple uses; 4) can be used in challenging, creative and uncertain ways; 5) provide interesting sensory experiences (e.g., from touch or movement); 6) any hazards inherent to the materials can easily be identified and managed by a child; and 7) are, or are made from, recycled materials. New materials will be introduced periodically to replace broken items and complement existing materials. Teaching staff will be asked not to intervene in the play unless children are at risk of imminent harm. Maintenance of the materials will be the responsibility of researchers in collaboration with staff from each program.

### Control phase

During the control period, the playground space will not be modified and standard play will continue. The same volume of playground observations will occur for both intervention and control periods.

### Playground observation system

During control and intervention periods, 3 to 5 days per week, we will record the activities and social interactions in which children and adults engage in the playground during lunch and recess periods. We use a purpose-built iPad application modified from the System for Observing Play and Leisure Activity in Youth (SOPLAY) [17]. We will train observers and check reliability against a gold standard observer. We will use these findings to calculate the primary outcome: quality of play and social interactions on the playground. This will be assessed by looking at the ratio of positive play transactions to time spent in negative play, through data collected from the SOPLAY observations. No observations will take place during rainy weather or at any other time when play is conducted indoors.

### Coping inventory [18]

An observation instrument used to assess the skills and resources a child uses to meet personal needs and adapt to the demands of the environment. The CI is a 48-item scale completed by teachers at the beginning and end of each study phase. Factor analysis revealed evidence for one factor accounting for 75 % of the variance; high correlation with school achievement (California Achievement Tests; *r =* .71); significant but low correlations with self concept (Piers Harris; *r* = .17); inter-rater reliability *r =* .90–.92 and internal consistency α–.84–.98; *SEm* ≈ .03 [18]. We have established evidence for test–retest reliability (intra-class correlation = .96).

### Tolerance of Risk in Play Scale (TRiPS) [19]

TRiPS is a 31-item measure regarding the activities that adults condone for children aged 2 to 12 years. Rasch analysis of Version 1 of TRiPS, developed for parents of typically-developing children, revealed excellent evidence for internal construct validity (goodness of fit within acceptable range for all items) and reliability (person separation 2.63; reliability index = 0.87) and near perfect correlation with child age [19]. TRiPs version 2 will be completed by parents and teachers at the beginning and end of the intervention phase.

### Adult Playfulness Trait Scale (APTS)

APTS is a 19-item self-report measure of adult playfulness. The APTS consists of three sub-dimensions: fun-seeking motivation (fun belief, initiative and reactivity), uninhibitedness and spontaneity. Multiple steps of conceptual evaluation have demonstrated adequate validity and reliability [20]. The APTS will be administered to staff before commencement of the intervention period.

### Video recording of uncertainty

The iPADs are used to video record any uncertain or ‘risky’ behaviours which occur on the playground. When children are viewed to be currently partaking in risky behaviour, or their behaviour looks like it could potentially become a risky situation, the children’s activities and behaviours are recorded on video. The video recording is performed by an unobtrusive member of the research team who does not interact with the children. The videos are then stored for future analysis, primarily looking at how the ‘risky’ situation is resolved (i.e. by an adult or by the child).

### Qualitative evidence

In-depth (~1 h), semi-structured interviews with parents and teachers nominated by principals are purposely selected to represent a range of opinions about the value of the program or tolerance of risk. Qualitative interviewing is an optimal way to understand the subjective experiences and world views of participants. We will adhere to accepted procedures to ensure trustworthiness of the data (e.g., triangulation. journaling) [21].

### Process evaluation

Researchers are present at the schools three to five days per week. In addition to observing the designated play space, they will have regular formal and informal conversations with school staff to ascertain the degree to which the intervention has been taken up (e.g., use of materials; teachers’ attitudes and beliefs) and monitor difficulties and particular successes.

### Outcome evaluation

Both quantitative and qualitative evaluation will occur. Quantitative: we will measure the effectiveness of the intervention for changing interactions and behaviours on the playground (primary outcome) and teacher’s tolerance for risk in play at the cluster level, as well as at an individual level. We will also measure children’s coping abilities, parents’ and staffs’ tolerance for risk in play and willingness to grant autonomy. We will use Wilcoxon signed-rank tests to compare net change from baseline values during intervention and non-intervention phases for cluster-level outcomes. We will analyse individual-level outcomes using multi-level modelling to examine the effect of the intervention, allowing for any period effect and adjusting for clustering by school. We will use intervention phase data for establishing the sustainability of the intervention and gains. We will compare outcomes at the end of the intervention with those at the end of the control phase for all six schools to test for within-school change, adjusted for clustering. Qualitative: We will conduct semi-structured interviews with four parents and two teachers from each program after they have been involved in the program one year. Adults will be selected purposively to represent a range of opinions about the value of the program. We will explore shifts in perceptions of risk, comfort with promoting manageable risk-taking and children’s abilities to assume responsibility. Data will be transcribed verbatim and subjected to constant comparative analysis to identify emergent themes according to Charmaz’s [22, 23] approach to social analysis.

### Economic evaluation

We will conduct an economic evaluation to compare the relative costs and outcomes associated with the intervention. A rigorous costing analysis will be undertaken, exploring the real world costs of implementing the intervention. This will include the small cost of providing the play materials and the larger cost of the risk-reframing sessions. The intention of doing this will be to estimate the financial implication of a broader roll-out of the approach to other similar schools, thereby supporting the translation of this novel research. For the cost-effectiveness analysis, the costs of providing the intervention will be contrasted with the incremental outcomes detailed previously (see Table 1), to provide some evidence for policy-makers to assess the feasibility of this interventional model. We do not believe that any benefit associated with the intervention would manifest in changing utility scores, hence we have not proposed a cost-utility analysis. We do however believe that the estimation of cost implications of the intervention forms a part of the appraisal of the approach.

## Discussion

New national programs, such as the National Disability Insurance Scheme, will provide support to people with disabilities by encouraging inclusion and access to mainstream activities and providing the framework to achieve their goals and aspirations. This initiative will allow people with disabilities to assume greater responsibility of their own daily lives. However, we need to ensure that young people with disabilities are suitably equipped to appropriately assume this responsibility that is expected later in life. Innovative approaches, such as the one we have proposed, are desperately needed, to prepare young people and their carers for a future of autonomy and independence. It is essential that the development of these skills is promoted in primary school aged children, to provide a foundation for life long attributes. This early intervention can occur by including children in age-appropriate ways and simultaneously addressing the issues of adults who have the most significant influence — parents and teachers. The research presented is an important step toward promoting choice and control for children with disabilities and by encouraging autonomy and responsibility may ultimately improve quality of life. If this research is shown to be feasible, efficacious and cost-effective, it may provide policy-makers with evidence to translate this model into standard practice in Australian schools.

## Abbreviations

APTS: adult playfulness trait scale
SOPLAY: system for observing play and leisure activity in youth
TRiPS: tolerance of risk in play
